# 

**Published:** 1890-09

**Authors:** 


### HALL'S JOURNAL OF HEALTH

ESTABLISHED 1854

CONTENTS.

Vol. 37. No. 9.

| | PAGE. |
|---|---|
| The Summer Outing | 193 |
| Beer as a Tonic | 194 |
| A Girl's Training | 196 |
| Is Crime a Disease | 197 |
| We Eat Too Much | 198 |
| Convulsions | 200 |
| Chemicals for Household Use | 200 |
| How Monkeys are Captured | 201 |
| Treatment of Sunstroke | 202 |
| Co-operative Housekeeping | 203 |
| Proper Clothing | 204 |
| Physical Culture | 205 |
| What Education is Doing for Us | 206 |
| Wonderful Coal Products | 206 |
| Sulphur in Sugar | 207 |
| Infant Foods | 208 |
| The Ear | 208 |
| Early History of the Saw | 209 |
| Miscellaneous | 210 |
| Preserving the Health | 210 |
| Boston Baked Beans | 211 |
| The Art of Bread Making | 212 |
| Salt for Moths | 213 |
| Ten Good Things to Know | 215 |
| Literary | 215 |
| A Wish on the Moon | 216 |

INDEX TO ADVERTISEMENTS OF HALF A PAGE AND OVER.

| | PAGE. |
|---|---|
| Woman's Work | I |
| Celluloid | II |
| Hollow Suppositories | III |
| The Esoteric | IV |
| Hospital Remedy Co. | IV |
| R. W. Shoppell | IV |
| Hall's Journal of Health | V |
| Goldsmith's Pianos | V |
| E. C. Morris & Co. | VI |
| Dennin's Certain Cure | VII |
| The Victor Safe and Lock Co | VII |
| Revised Premium List | VIII |
| Rochester Lamp Co | IX |
| Herba-Vita Remedy Co | X |
| Christ Before Pilate | XI |

